# Coexistence of diploid, triploid and tetraploid crucian carp (Carassius auratus) in natural waters

**DOI:** 10.1186/1471-2156-12-20

**Published:** 2011-01-29

**Authors:** Jun Xiao, Tuomi Zou, Yubao Chen, Lin Chen, Shaojun Liu, Min Tao, Chun Zhang, Rurong Zhao, Yi Zhou, Yu Long, Cuiping You, Jinpeng Yan, Yun Liu

**Affiliations:** 1 Key Laboratory of Protein Chemistry and Fish Developmental Biology of Education Ministry of China, College of Life Sciences, Hunan Normal University, Changsha 410081, China

## Abstract

**Background:**

Crucian carp (abbreviated CC) belongs to the genus of *Carassius *within the family of *Cyprinidae*. It has been one of the most important freshwater species for Chinese aquaculture and is especially abundant in the Dongting water system of Hunan province. CC used to be considered as all diploid forms. However, coexistence of diploid (abbreviated 2nCC), triploid (abbreviated 3nCC) and tetraploid crucian carp (abbreviated 4nCC) population of the Dongting water system was first found by our recently researches.

**Results:**

We examined the ploidy level and compared biological characteristics in different ploidy CC. In reproductive mode, 2nCC was bisexual generative and 4nCC generated all-female offspring by gynogenesis. However, 3nCC generated progenies in two different ways. 3nCC produced bisexual triploid offspring fertilized with 3nCC spermatozoa, while it produced all-female triploid offspring by gynogenesis when its ova were activated by heterogenous spermatozoa. The complete mitochondrial DNA of three different ploidy fishes was sequenced and analyzed, suggesting no significant differences. Interestingly, microchromosomes were found only in 3nCC, which were concluded to be the result of hybridization. Allogenetic DNA fragments of Sox genes were obtained in 3nCC and 4nCC, which were absent in 2nCC. Phylogenetics analysis based on Sox4 gene indicated 3nCC and 4nCC formed a separate group from 2nCC.

**Conclusions:**

In summary, this is the first report of the co-existence of three types of different ploidy crucian carps in natural waters in China. It was proved that the coexistence of different ploidy CC was reproductively maintained. We further hypothesized that 3nCC and 4nCC were allopolyploids that resulted from hybridization. The different ploidy CC population we obtained in this study possesses great significance for the study of polyploidization and the evolution of vertebrates.

## Background

Taxonomically, crucian carp (*Carassius auratus *L., abbreviated CC) belongs to the genus of *Carassius *within the family of *Cyprinidae*. It is widely bred across Eurasia and America, because of its good survival rate, high reproduction rate and good disease resistance. In China, the species has been found in most provinces, except for the western plateau. It is one of the most important freshwater species for Chinese aquaculture especially in the Dongting water system of Hunan province. Although *Carassius auratus gibelio *(superspecies *auratus*) has been reported co-existence of three types of different ploidy [1-4], CC (*Carassius auratus*) used to be considered as all diploid forms in China [5,6]. However, triploid crucian carp has been reported in China since '80 s, including Dianchi High-back crucian carp in Yunnan province [7], the Sogu crucian carp in Guangdong province [8], Puan crucian carp in Guizhou province [9], and Pengze crucian carp in Jiangxi province [10] et al. In this study, CC individuals were sampled from the Dongting water system of Hunan province and assayed by flow cytometry and chromosome counts. The results confirmed that there were three forms with different ploidy in the natural water system. It is the first report of tetraploid forms in a natural CC population of China (not *Carassius auratus gibelio*). Meanwhile, we believed that polyploidization in crucian carp is a process in evolution. This research aims to approach the reason why there is the same polyploidzation in crucian carp in China as in *Carassius auratus gibelio*.

Polyploidy is defined as organisms with one or more additional chromosome sets. It has been widely recognized in plants, such that about 30-70% plants are polyploidy in their evolutionary history [11]. So far, some researches have shown that polyploidy is not only a characteristic of plants, but also occurs in other eukaryotes [12,13]. The polyploid event(s) possibly happened in the evolution of prokaryotes to humans [14]. In '70 s, Ohno proposed the theory that two rounds of tetraploidization occurred in the evolution of vertebrates [12,15,16]. The third genome duplication, termed fish-specific genome duplication, was also reported in teleosts. So far, over 28,000 fish species have been identified, which is more than that of all the other vertebrate groups combined. Recent studies on comparative genomics have further suggested that the third round of fish-specific genome duplication might have occurred in ray-finned (actinopterygian) fishes about 350 million years ago during the divergence between teleost fish and basal actinopterygian lineages. Polyploidy induces redundant genes, increasing the possibility of gene loss, gene silencing, subfunctionalization of genes, and evolution to new genes [17-21]. These variations provide polyploids with more possibilities for new characteristics and adaptations than diploids. Therefore, polyploidy might be related to the increase in species number and biological diversity [22-26]. In addition, it seems that the duplication of fish genome may be still in progress in the nature [27]. In general, a species has definite chromosome numbers. However, in this study, the wild CC population consisting of three forms with different ploidy provided excellent material for the studies on polyploidization. Could the ploidy diversity of the population maintain? How did the polyploidy level change?

The duplicated sets of chromosomes originate from the same or a closely related individual ("autopolyploid") or from the hybridization of two different species ("allopolyploid"). This observation has traditionally led to the conclusion that autopolyploids are ephemeral, whereas allopolyploids give rise to the majority of long-lasting lineages. In our previous study, two kinds of artificial polyploids were established using distant hybridization. We have reported artificial allotetraploid hybrids of red crucian carp (*Carassius auratus*) × common carp (*Cyprinus carpio*), which is the first case of the creation of a bisexual fertile allotetraploid population in fish (perhaps even the first in vertebrates) [28]. Subsequently, the polyploid hybrids from different subfamilies of fish, red crucian carp (*Carassius auratus*) × blunt snout bream (*Megalobrama amblycephala*), were also obtained [29]. Then, what was the possible origin of the polyploid forms in the CC population in nature waters?

We have been continually monitoring the CC population since 2005. The fertility of different ploidy CC was studied. The coexistence of three different ploidy individuals in the wild CC population was proved to be reproductively stable to some extend. The present study focused on the characteristics of different ploidy fish from natural waters, with the purpose of investigating the origin of the rules for natural polyploidy and illuminating the roles of polyploidization in the evolution of fish or vertebrates.

## Results

### DNA content

**Figure 1:**
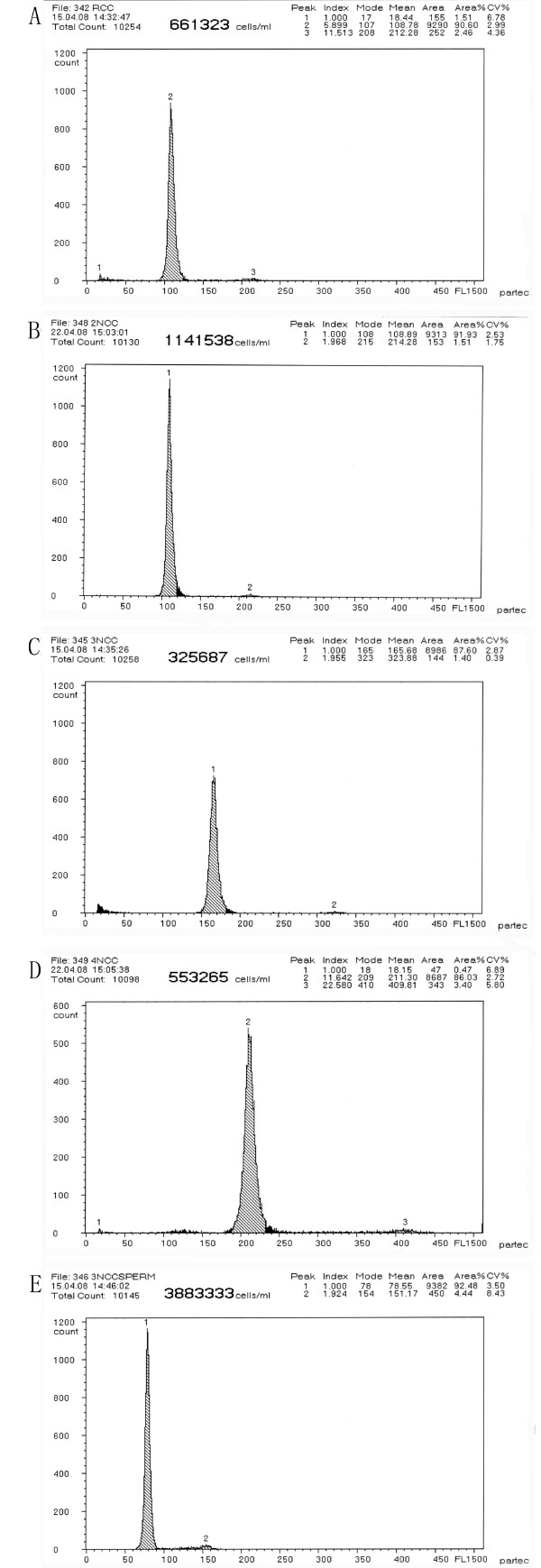
**Cytometric histograms of DNA fluorescence for different ploidy CC**. (A) The mean DNA content of RCC (peak 2:108.78, area%: 90.60). (B) The mean DNA content of 2nCC (peak 1:108.89, area%: 91.93). (C) The mean DNA content of 3nCC (peak 1:165.68, area%: 87.60). (D) The mean DNA content of 4nCC (peak 2:211.31, area%: 86.03). (E) The mean DNA content of 3nCC sperm (peak 1:78.55, area%: 92.48).

A flow cytometer (Partec GmbH) was employed for DNA content measurement in large scale. All sampled CC were collected from wild population and all the progenies obtained in the breeding test were detected in this way. The distribution of DNA content of each sample is shown in Table 1 and Figure 1. The DNA content of red crucian carp (2n = 100, abbreviated RCC) was used as a control. The samples with mean DNA content that was equal (*P *> 0.05) to that of RCC were determined as diploid (abbreviated 2nCC) with two sets of chromosomes (2n = 100) similar to RCC. The individuals that possessed the mean DNA contents of 1.5 times, 2 times (*P *> 0.05) to that of RCC were determined respectively as triploid (abbreviated 3nCC) and tetraploid (abbreviated 4nCC) which contained three (3n = 150) and four (4n = 200) sets of chromosomes. The mean DNA content of 3nCC sperm was 0.75 times to that of RCC, which was half of that of 3nCC blood cells, indicating the sperm contained 0.75 set of chromosomes (0.75n = 75). We examined 949 CC in past five years. 2nCC, 3nCC and 4nCC were found in the natural CC population of the Dongting water system, accounting for 16%, 80%, and 4%, respectively. The number of 4nCC was much fewer than 2nCC. The percentage of 2nCC decreased roughly year by year, account for 39%, 23%, 20%, 3% and 5%, respectively. The percentage of 3nCC increased roughly year by year, accounting for 61%, 68%, 74%, 97% and 94%, respectively (Table 2).

**Table 1:** Mean DNA content in RCC, 2nCC, 3nCC, and 4nCC

Fish type	Mean DNA content	Ratio Observed	Expected
RCC	108.78		
2nCC	108.89	2nCC/RCC = 1^1^	1
3nCC	165.68	3nCC/1.5RCC = 1.02 ^1^	1
4nCC	211.31	4nCC/2RCC = 0.97 ^1^	1
3nCCSPERM	78.55	3nCCSPERM/0.75RCC = 0.96 ^1^	1

^1 ^The observed ratio was not significantly different (*P *> 0.05)

**Table 2:** The quantity and gender diversity of different ploidy CC detected in the past five years

Year	Total	2nCC		3nCC		4nCC	
		Number	Ratio of the population	Number	Ratio of the population	Number	Ratio of the population
2005	77	30 (17 ♀, 13♂)	39%	47 (45 ♀, 2♂)	61%	0	0%
2006	271	61 (33♀, 28♂)	23%	186 (164♀, 22♂)	68%	24 (24♀, 0♂)	9%
2007	225	46 (22♀, 24♂)	20%	166 (134♀, 32♂)	74%	13 (13♀, 0♂)	6%
2008	237	8 (2♀, 6♂)	3%	229 (170♀, 59♂)	97%	0	0%
2009	139	7 (1♀, 6♂)	5%	130 (104♀, 26♂	94%	2(2♀, 0♂)	1%

### Chromosome numbers

**Figure 2:**
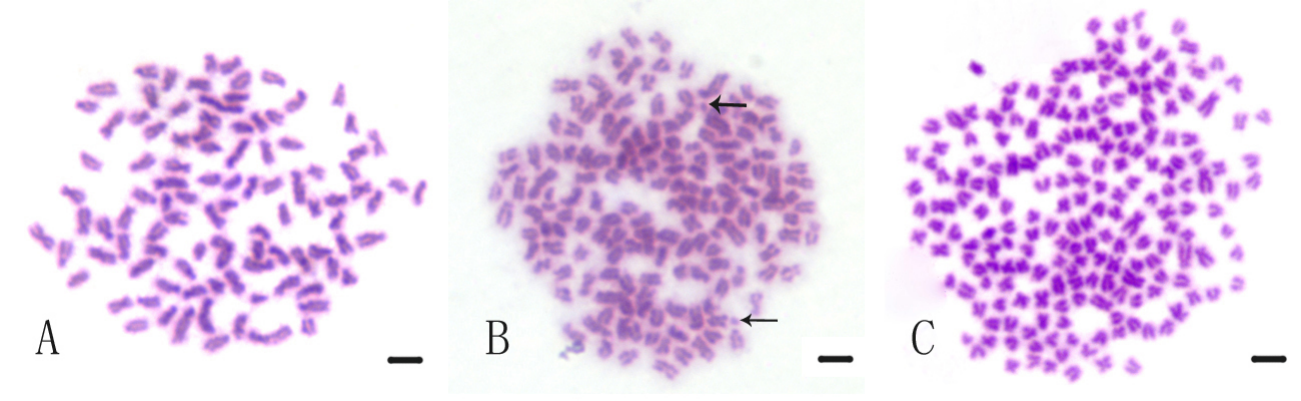
**Chromosome spreads at metaphase in 2nCC, 3nCC, and 4nCC**. (A) The metaphase chromosome spreads of 2nCC (2n = 100); (B) The metaphase chromosome spreads of 3nCC (3n = 150), in which some uncounted minute chromosomes (arrows) are indicated; (C) The metaphase chromosome spreads of 4nCC (4n = 200). Bar in A-C, 4 μm.

To determine the ploidy accurately, chromosome counts were done on kidney tissue of samples. Figure 2 showed examples of the metaphase chromosome spreads in the three types of CC with different ploidy. Table 3 presented the distribution of chromosome numbers in 2nCC, 3nCC and 4nCC. Of all the examined samples in 2nCC, 96% of chromosomal metaphases possessed 100 chromosomes, proving that they were diploids with 100 chromosomes (2n = 100) (Figure 2A). 4% of chromosomal metaphases with less than 100 chromosomes were acceptable considering the low probability of chromosome losing during experiment operation. Of all the examined samples in 3nCC, 94% of chromosomal metaphases had 150 chromosomes, indicating that they were triploids with 150 chromosomes (3n = 150) (Figure 2B). Several uncounted particles resembling chromosomes (Figure 2B, arrows) in 3nCC were identified as microchromosomes. Of all the examined samples in 4nCC, 71% of chromosomal metaphases had 200 chromosomes, indicating that they were tetraploids with 200 chromosomes (4n = 200) (Figure 2C).

**Table 3:** Examination of chromosome number in 2nCC, 3nCC, and 4nCC

Distribution of chromosome number							
Fish type	No. in metaphase	<100	100	<150	150	<200	200
2nCC	200	9	191				
3nCC	200			13	187		
4nCC	200					59	141

### Morphological traits and gender diversity

To detect the appearance diversity in the population, 15 individuals of each ploidy form that were previously determined by DNA content were random sampled for morphologically examining. The three different ploidy forms of CC were similar in appearance and no significant differences in morphological traits were found (P > 0.05). Table 4 and Table 5 detailed their examined measurable traits and countable traits (more details about weight, measurable traits and countable traits were in additional file 1, additional file 2, additional file 3, additional file 4, additional file 5 and additional file 6). The gender diversities of different ploidy CC detected in the past several years were counted (Table 2). In this study, both the males and females were found in 2nCC with the sex ratio (female: male) of 1:1. We also found males and females in 3nCC population but the sex ratio (female: male) was nearly 5:1. Until now, only females were found in 4nCC, and the number of 4nCC in the population was apparently less than 2nCC and 3nCC. To some extent, the gender diversities of different ploidy CC indicated that the reproduction mode of 3nCC and 4nCC may not be amphigenesis.

**Table 4:** Measurable traits of 2nCC, 3nCC, and 4nCC

Fish type	WL/BL	BL/BW	BL/HL	HL/HW	BW/HW	TL/TW
2nCC (2n = 100)	1.24 ± 0.05	2.72 ± 0.20	3.75 ± 0.27	1.06 ± 0.12	1.47 ± 0.15	0.93 ± 0.14
3nCC (3n = 150)	1.24 ± 0.03	2.76 ± 0.10	3.90 ± 0.24	1.09 ± 0.06	1.53 ± 0.11	0.95 ± 0.10
4nCC (4n = 200)	1.22 ± 0.04	3.06 ± 0.26	4.10 ± 1.23	1.12 ± 0.18	1.44 ± 0.14	1.02 ± 0.18

**Table 5:** Countable traits of 2nCC, 3nCC, and 4nCC

Fish type	Lateral scales	Upper lateral scales	Lower lateral scales	Dorsal fins
2nCC (2n = 100)	27-30	5-7	6	III+16-18
3nCC (3n = 150)	27-29	6	6	III +17-19
4nCC (4n = 200)	27-29	6	6	III +17-19

### Fertility analysis

**Figure 3:**
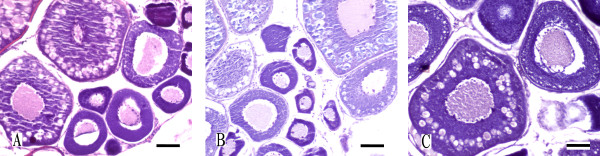
**The ovarian microstructure of different ploidy CC**. (A) The ovarian microstructure of 2nCC. (B) The ovarian microstructure of 3nCC. (C) The ovarian microstructure of 4nCC. Bar in A-C, 0.02 mm. As the figure shows, the ovaries of three forms of CC with different ploidy entered III-stage, indicated that the females of three kinds of CC were fertile.

Observation of the gonad microstructure and ultra-structure indicated the fertility of different ploidy CC. The ovarian microstructure under section observation indicated that the females of three kinds of CC were fertile. The ovarian development of the population was normal and synchronous (Figure 3). The ovaries of three forms of CC with different ploidy entered III-stage, where II-stage oocytes and III-stage oocytes were simultaneously found.

**Figure 4:**
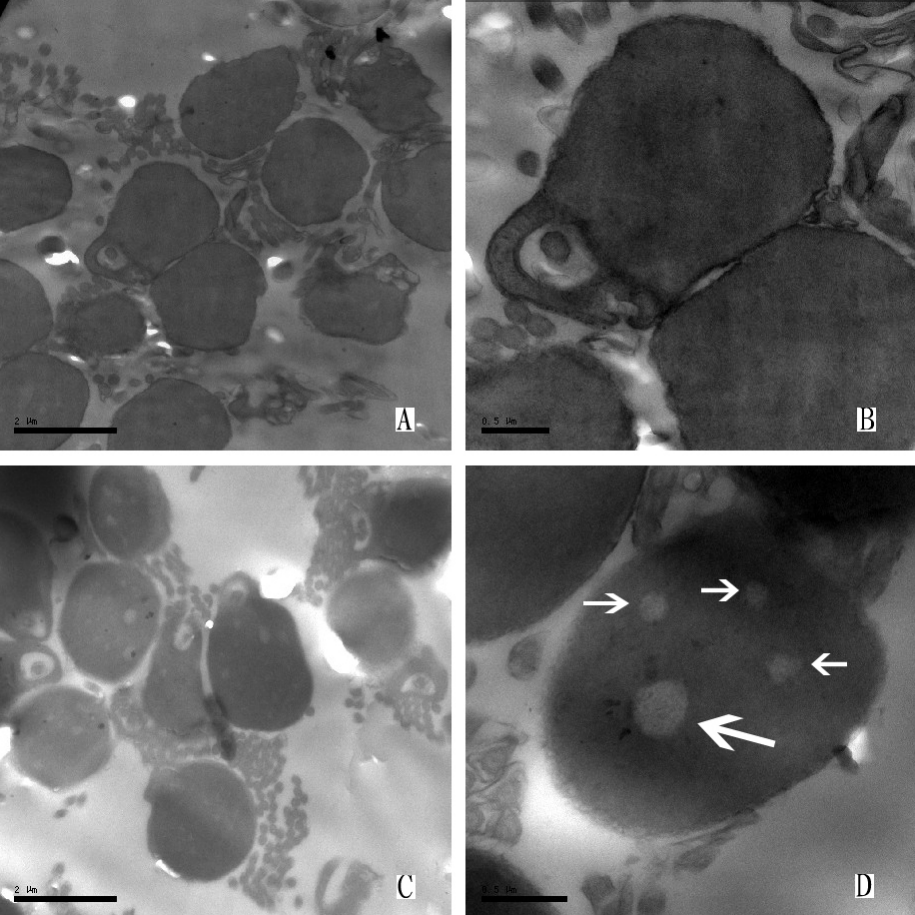
**The testis ultra-structure of 2nCC and 3nCC**. (A) and (B), the mature testis of 2nCC. (C) and (D), the mature testis of 3nCC, the vacuoles (arrow) are indicated. Bar in part A and C, 2 μm; in part B and D, 0.5 μm. All the mature spermatozoa in the testis of 2nCC and 3nCC had normal structures, indicated that 2nCC and 3nCC were also fertile. In the testis of 3nCC there were certain amounts of vacuoles in the head of the spermatozoa.

**Figure 5:**
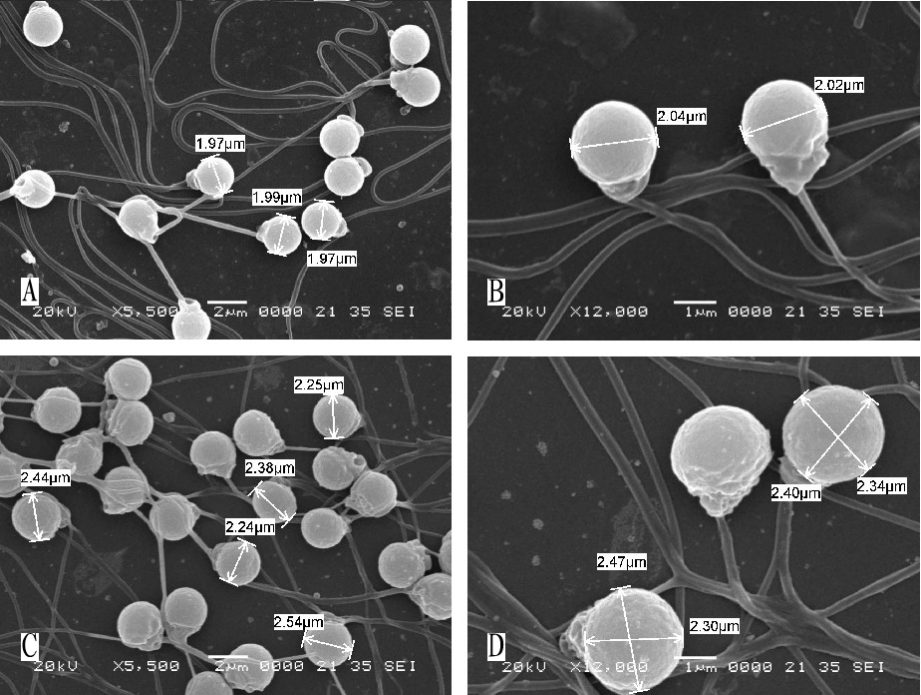
**The mature spermatozoa of CC**. (A) and (B), the mature spermatozoa of 2nCC. (C) and (D), the mature spermatozoa of 3nCC. Bar in part A and C, 2 μm; in part B and D, 1 μm. As the figure shows, the mature spermatozoa of 2nCC and 3nCC had the normal external form, comprising a head, a connecting piece, and a tail.

Males were found in 2nCC and 3nCC. The sperm ultra-structure indicated that 2nCC and 3nCC were also fertile. Under TEM observation, all the mature spermatozoa in the testis of 2nCC and 3nCC had normal structures (Figure 4 A and 4B). However, in the testis of 3nCC there were certain amounts of vacuoles in the head of the spermatozoa (Figure 4 C and 4D, arrow), the biological significance of which was not clear and will require further study. Under SEM observation, the mature spermatozoa of 2nCC and 3nCC had the normal external form, comprising a head, a connecting piece, and a tail (Figure 5). With the increase of ploidy level, the head size of spermatozoa increased (Table 6). The mean head size of spermatozoa ratio of 3nCC to 2nCC was 1.5:1(*P *> 0.05), the same as the DNA content.

**Table 6:** Head volume of spermatozoa of 2nCC and 3nCC

			Volume ratio	
Fish type	diameter(μm)	**Volume(μm**^**3**^**)**	Observed	Expected
2nCC	2.00 ± 0.05	4.19 ± 0.25		
3nCC	2.32 ± 0.22	6.53 ± 2.04	3nCC/1.5*2nCC = 1.04^1^	1

^1 ^The observed ratio was not significantly different (*P *> 0.05) from the expected ratio.

### Breeding tests

The reproduction mode of different ploidy CC can be inferred through the study of the breeding-tests-produced progeny's biological characters. All the crossings had high fertilization rate (>70%) and hatching rate (>60%), and produced more than 300 living offspring, except the crossing of 2nCC♀ × BSB♂ which possessed low fertilization rate (46%) and hatching rate (17%) and produced 119 living hybrids. We believed that the further relationship between 2nCC and BSB (belonged to different subfamily) was the major reason of the lower fertilization rate and hatching rate (more details about fertilization rate and hatching rate were in additional file 7).

The offspring produced in each crossing were separately bred. Their morphological traits, gonad development and chromosome numbers were detailed in table 7. Within the population of CC, the eggs of 2nCC accepted only the sperm of 2nCC and developed into diploid progenies. When fertilized with active sperm coming from related species out of the population, the progenies of 2nCC possessed hybrid phenotypes and delayed development of gonads. The eggs of 2nCC could not be activated by UV-inactivated sperm.

**Table 7:** Morphological traits, gonad development, and chromosome numbers of the progeny

		intraspecies		intergenus	intersubfamily	
		2nCC (♂)	3nCC (♂)	Common carp (♂)	BSB (♂)	Deactivated sperm
2nCC (♀)	morphology	Like CC	death	hybrid	hybrid	death
	gonadal development	Normal, bisexual		delayed	delayed	
	chromosome number	100		100	148	
3nCC (♀)	morphology	Like CC	Like CC	Like CC	Like CC	Like CC
	gonadal development	Normal, all-female	Normal, bisexual	Normal, all-female	Normal, all-female	Normal, all-female
	chromosome number	150	150	150 200	150	150
4nCC (♀)	morphology	Like CC	Like CC	Like CC	Like CC	Like CC
	gonadal development	Normal, all-female	Normal, all-female	Normal, all-female	Normal, all-female	Normal, all-female
	chromosome number	200	200	200	200	200

The eggs of the all-female 4nCC only could develop into all-female progenies with similar phenotypes to the maternal parent, when the eggs were fertilized with the sperm from a paternal within or without the CC population, and even with UV-inactivated sperm. All the progenies of 4nCC were detected as tetraploids with a chromosome number of 200.

Interestingly, the bisexual 3nCC was fertile. When it fertilized with homogenous sperm of 3nCC, the eggs of 3nCC developed into bisexual triploid progenies with normally developed gonads. In this cross, bisexual offspring were produced. However, when fertilized with heterologous sperm of 2nCC or from other species, the eggs of 3nCC developed into all-female triploid progenies with similar appearance to the maternal 3nCC. There were no male offspring produced. In the cross of triploid males with triploid females, male offspring were produced. It obviously indicated that the male offspring obtained at least the male gene from the triploid males. The only exception was discovered in the cross 3nCC (♀) × common carp (♂). There were not only a large number of all-female triploid forms (3n = 150) but also several tetraploid forms (4n = 200) (frequency 0.3%) in the progenies. These two kinds of progenies possessed similar phenotypes. The only way to distinguish them was to detect their DNA content or chromosome spreads. The tetraploid forms were all female with normally developed gonads and did not exhibit hybridized phenotypes.

### DNA-bands of the HMG-box of Sox genes

**Figure 6:**
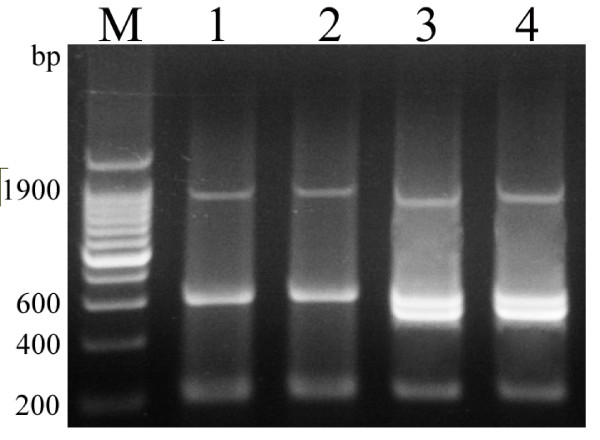
**The DNA-band patterns of Sox genes. M, DNA ladder with an increase of 200bp**. 1. RCC; 2. 2nCC; 3.3nCC; 4.4nCC.

The Sox gene is karyogene. If peregrinus chromosome is integrated, the recombination can be observed. There were three Sox DNA fragments (about 200, 600 and1900bp) in RCC and 2nCC, and four DNA fragments (about 200, 500, 600 and 1900bp) in 3nCC and 4nCC (Figure 6). 3nCC and 4nCC possessed an extra 500bp band that did not belong to 2nCC, the original member of the CC population.

**Figure 7:**
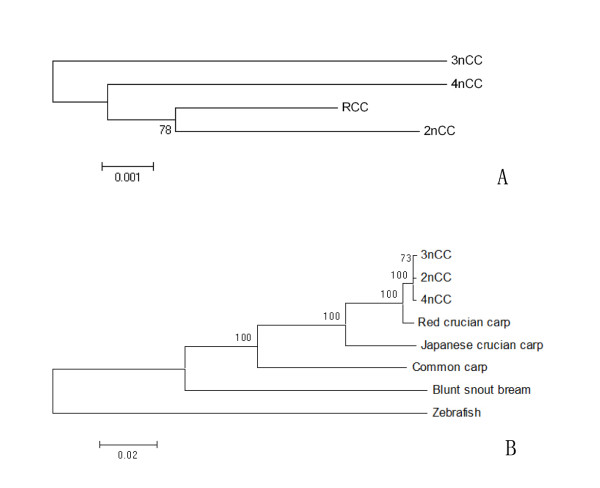
**Phylogenetic study using the 1900bp Sox-HMG fragments and the complete mt-DNA sequences**. (A) The NJ dendrogram depicts the relationships among 1900bp fragment in CC. (B) The NJ dendrogram depicts the relationships among complete mt-DNA sequence in these fishes.

The exact lengths of about 1900bp fragments of 2nCC, 3nCC and 4nCC were 1941bp, 1943bp and 1955bp respectively and submitted to GenBank under accession nos. [GenBank: GU294783, GenBank: GU294782, GenBank: GU294784] respectively. Sequence homology and variation were analyzed by CLUSTALW 2.0 (details of the analysis were in additional file 8). Till now, the 1900-bp Sox-HMG fragments were only found in the fish belonging to the genus of *Carassius*. The phylogenetic tree (Figure 7A) of 1900-bp Sox-HMG fragments indicated that the relationship between 2nCC and red crucian carp was closer than that among the CC population, and that 3nCC and 4nCC were comparatively distant-related to 2nCC.

### The complete mitochondrial genome sequences

The mitochondrial DNA strictly abides by maternal inheritance. Through the study of the mt-DNA, the maternal parent of different ploidy CC can be indicated. The complete mt-DNA sequences of 2nCC (16580bp), 3nCC (16580bp) and 4nCC (16581bp) were obtained and submitted to GenBank under accession nos. [GenBank: GU086395, GenBank: GU086396, GenBank: GU086397] respectively.

The phylogenetic tree performed by bootstrap in neighbor joining with the complete mtDNA sequences was shown in Figure 7B. The grouping matches the taxonomy. The tree firstly diverges into clades of different subfamily: the *Cultrinae *subfamily including the blunt snout breams and the *Cyprininae *subfamily containing the others which were supported by high bootstrap values. The clade of *Cyprininae *subfamily has two groups of different genus: one including the common carp of genus *Cyprinus*, and the other belonging to the genus of *Carassius*. The three different ploidy CC clustered together indicating that the CC population had close genetic relationship.

## Discussion

Fish possessed the greatest variety in vertebrates and widely spread all over the world. Then, it is very interesting and valuable to study how fish could evolve so successfully in species forming and environment adoptability. Polyploidization is regarded as the important characteristic of genome evolution of all eukaryotes, which possibly has occurred in the evolutionary progress of protozoa and human beings. Fish-specific genome duplication was also reported in teleosts [18,19,30]. Polyploidy induces genome multiplication, resulting in redundant genes that provide genetic basic for specie evolution at the molecular level. Thus, frequent polyploidization may relate to the higher species variety of fish and other organisms [22-26]. In this study, our interesting discovery and convincing experiments provided an extraordinary insight to further consideration on fish polyploidization.

Crucian carp is a traditional economic freshwater fish and is widespread in China. The ploidy of CC population which was well-focused for years used to be considered as all diploid forms. However, some reports have discovered triploid forms in population of wild CC in China since 1980s [7-10]. Since then, the co-existence of diploid and triploid forms in natural CC populations has become widely accepted. In this study, by using flow cytometry to examine the DNA content of erythrocyte nuclei, the high diversity of CC was simply and accurately disclosed at the genome level. It was extremely interesting that there were not only diploid form (2n = 100) and triploid form (3n = 150+), but also tetraploid form (4n = 200) were discovered for the first time in a natural CC population in China.

### Reproductive mode and chromosome set

According to our continual detection of the wild CC population since 2005, the coexistence of polyploid individuals in wild CC population was stable to some extend, although the detected proportions of each ploidy group changed every year. The polyploid forms that took up a certain proportion were not short-lived but persistent. How did the triploid and tetraploid forms maintain in the wild population? The series of crossing tests provided valuable information. The reproductive modes revealed that 2nCC were identified as the normal bisexual group, producing haploid gametes that developed into new diploid generation when fertilized with the sperm of 2nCC. The all-female 4nCC were considered as the natural gynogenetic reproductive mode. The morphology, gonadal development and chromosome number of 4nCC offspring were examined. They all showed the same genotypes as their maternal parents. We didn't obtain male tetraploid crucian carp from nature in five years. Meanwhile, in the crosses of female 4nCC with male fishes of different relationship, tetraploid offspring which were all-female with the same morphology and chromosome number as their maternal parents were obtained, which by hybridization couldn't be obtained. The eggs of 4nCC would develop into new tetraploid progenies, no matter what spermatozoa of different species they were fertilized with. Natural gynogenesis was supported by the results of morphological analysis, chromosome tests and gonad development observation. The spermatozoa only activated the eggs' development, the chromosomes of which did not contribute to the zygote. The reproductive mode of 3nCC was complex. As mentioned above, the 3nCC were bisexual fertile. When the eggs of 3nCC met the spermatozoa within the group, bisexual triploid offspring were generated, suggesting that 3nCC possessed normal bisexual reproduction (perhaps, 2n = 150), similar to 2nCC. In contrast, when the eggs of 3nCC met heterogenous spermatozoa of allied species, all-female offspring were produced, suggesting that 3nCC was also capable of natural gynogenesis reproduction, as 4nCC was. Actually, it is traditionally considered, and widely accepted, that triploids are sterile. Because the chromosomes of the germ cells cannot be paired correctly, abnormal gametes were induced in the first meiosis. It seems difficult to understand how the chromosomes of 3nCC performed pairing during meiosis. Considering the fish-specific genome duplication that was regarded as having happened in the history of teleosts evolution [17-20], 3nCC could actually be regarded as an ancient hexaploid which could certainly perform pairing of homologous chromosomes during meiosis. Therefore, the results supported the occurrence of genome duplication in fish evolution. In our opinion, the triploid crucian carp has diploidized to some extent and this is still in process in evolution. Thus, the polyploid forms of CC were not incidental, but were reproductively maintained in the wild population.

Moreover, potential competitive inhibition of 3nCC on 2nCC was indicated. The special reproductive mode of 3nCC provided them with great advantages. In the natural population of CC, the different ploidy forms lived in the same watercourse and copulated together within the same mating period. The eggs of 3nCC could accept sperms from both 2nCC and 3nCC and produce normal triploid progenies. In contrast, the eggs of 2nCC would develop into living offspring only when fertilized with the sperm of 2nCC. Thus, the existence of 3nCC would effectively inhibit the reproduction of 2nCC. We considered it as the reason for the substantial decrease of the proportion of 2nCC in the wild population, and speculated that the decreasing trend of 2nCC would accelerate together with the increasing 3nCC proportion. 2nCC would have even disappeared in the population in a certain period. The results indicated that it is necessary and vital for us to protect the wild CC species resources.

The extraordinary flexibility in the reproductive biology of triploids, and the apparent advent of gynogenesis in tetraploids represented a remarkable opportunity to study the evolutionary biology and formation of aneuploidy.

### Polyploidization in fish evolution

The fertility of 3nCC supported the occurrence of genome duplication in fish evolution at genome level. It is traditionally considered, and widely accepted, that triploids are sterile. Because the chromosomes of the germ cells cannot be paired correctly, then abnormal gametes were induced in the first meiosis. However, the 3nCC analyzed in this study were fertile and had normal, functional gonads that produced normal meiotic gametes. The DNA content of 3nCC's sperm was detected as half of that of the somatic cell, indicating that 3nCC had broken through the anticipated barrier and performed diploidization to a certain degree (perhaps, 2n = 150). It seems difficult to understand how the chromosomes of 3nCC performed pairing during meiosis. Considering the fish-specific genome duplication that was regarded as having happened in the history of teleosts evolution, 3nCC could actually be regarded as an ancient hexaploid which could certainly perform pairing of homologous chromosomes during meiosis [17-20]. Therefore, the results supported the occurrence of genome duplication in fish evolution.

The discovery of stable existence of tetraploid individuals in the original diploid wild population suggested a "new" tetraploidization that might be still in process in natural CC population. Besides, tetraploidy might not be directly resulted from duplication of diploid individuals, while triploid forms played a role in the ploidy diversity of the CC population. According to our breeding tests, the 4nCC only generated offspring by natural gynogenesis. Even when fertilized with the sperms of 2nCC, eggs of 4nCC still produced natural gynogens and no triploid progeny would be produced. However, in the crossing of 3nCC (♀) × common carp (♂), we obtained a few tetraploid individuals (4n = 200) (frequency 0.3%). It seemed that a complex of different ploidy forms was a possible intermediate phase in the process of tetraploidization.

The theory that two rounds of tetraploidization occurred in the evolutionary process of the vertebrates is popular [12,15,16]. Molecular evidence supporting the process of polyploidization mainly came from the multicopy of genes. At genome level, Venkatesh investigated the chromosome numbers of more than 300 fish species and found that all the chromosomal numbers were even and were approximately multiples of two different species, indicating that tetraploidization has occurred in the evolution of fishes [30]. However, it is quite difficult to find a "new" polyploidization to support the theory, because the assumed duplication events occurred millions of years ago. Our discovery of three different ploidy forms in a natural fish population suggested that genome duplication in fish might be still in progress and would spontaneously take place in nature.

### Hybridization, the possible origin of polyploid crucian carp

Furthermore, we considered the forming of polyploid CC attributable to hybridization.

Firstly, evidence came back from the observation of chromosomal metaphases of 3nCC. In the genome of 3nCC, some chromosome-like particles were found and identified as microchromosomes (supernumerary chromosomes or B-chromosome), in addition to the 150 standard chromosomes. These were found only in 3nCC, and there was no obvious pattern to their frequency of occurrence or amount. The origin of microchromosomes has been proved as interspecies hybridization in the gynogenetic fish *Poecilia Formosa*, a hybrid species between *P. mexicana *and *P. latipinna *[31]. This unisexual species required sperms of sexual parental species to initiate egg development, but paternal chromosomes are eliminated from the developing zygote. Laboratory crosses of *P. Formosa *(♀) and black strain (♂), both of which lacked microchromosomes, produced some black-pigmented offspring with microchromosomes. It was most likely that incomplete elimination of paternal normal chromosomes caused the appearance of microchromosomes (containing the paternal pigmentation genes) in the offspring [32]. Similar results were found for interspecific crosses between *Coix aquaticus *and *C. gigantean *[33]. Accordingly, the microchromosomes in 3nCC were considered as evidence of the heterogenous genome of related species, which was brought in through hybridization. Additionally, it was also reported that the existence and frequency of microchromosomes were related to the sex ratio [34]. In general, it is believed that microchromosomes play an important role in biological adaptability. For example, *Poecilia formosa *was found as a kind of unisexual species whose eggs required sperms of a sexual parental species to be activated but paternal chromosomes were eliminated from the developing zygote. It was believed that some paternal genetic material was incorporated into the zygote by the microchromosomes, which also played a role in the formation of body color [32]. Considering the reproduction of the 3nCC in the wild CC population, the microchromosomes might maintain a gene introgression within the population and reduce their reproductive isolation.

Secondly, stable coexistence of polyploid forms in the wild population also suggested the heterogenous origin, even though autopolyploidization could not be absolutely excluded. Autopolyploids, whose chromosomes were directly duplicated, were considered ephemeral, whereas allopolyploids gave rise to long-lasting lineages [26]. Accordingly, The Dianchi triploid High-back Crucian Carp of Yunnan province was not regarded as a direct duplication of the diploid Low-back Crucian Carp [35]. In this study, 4nCC maintained their all female and tetraploid nature in the progenies, with identical chromosome number and phenotypes, indicating a long-lasting linage of tetraploid forms in the CC population.

The reproductive mode of gynogenesis also suggested the heterogenous origin of 4nCC. Our previous studies have strongly supported the view that distant hybridization could result in polyploidization and produce hybrid progenies with a reproductive mode of gynogenesis. In the previous study [28,29], we established two kinds of artificial polyploidy lineage by hybridization. One was the cross of RCC (♀) × Common carp (♂). The F_1 _and F_2 _hybrids of RCC (♀) × Common carp (♂) were diploid hybrids with 100 chromosomes. Interestingly, the males and females of diploid F_2 _hybrids were able to generate unreduced diploid spermatozoa and diploid eggs, which were fertilized to form the allotetraploid hybrids in the F_3_. The diploid sperm and eggs of F_3 _were fertilized to produce the tetraploids in F_4_. Since then, the tetraploidy has been maintained and the population of F_3_-F_18 _tetraploid hybrids (abbreviated 4nAT) has been formed in succession. This was the first case of artificial creation of bisexual fertile allotetraploid hybrids in fish (maybe even in vertebrates). Then, the diploid gynogenetic hybrids and the diploid androgenetic hybrids were obtained, which developed from the diploid hybrid eggs and diploid sperm generated by the females and males of the tetraploid hybrids respectively, without the treatment for doubling the chromosomes [36,37]. Another artificial polyploid lineage was established by distant hybridization of RCC (♀) × blunt snout bream (♂). In this hybridization, the maternal fish, RCC, possessed 100 chromosomes and the paternal fish, blunt snout bream, possessed 48 chromosomes, and they belonged to different subfamilies (*Cyprininae *subfamily and *Cultrinae *subfamily). In the first generation of RCC (♀) × blunt snout bream (♂), we successfully obtained sterile triploid hybrids (3n = 124, abbreviated as 3nRB) and bisexual fertile tetraploid hybrids (4n = 148, abbreviated as 4nRB). The females of the 4nRB were also able to generate unreduced tetraploid eggs. Other researchers also obtained gynogenetic tetraploids by distant crossing [38-40]. It seemed that gynogenesis was more compatible for hybrids reproduction. In this study, 4nCC possessed natural gynogenesis. 3nCC would also produced gynogens when the eggs were fertilized with distant sperm. It was feasible and reasonable to infer the hybrid origin of polyploid CC.

Thirdly, the most powerful evidence came from the molecular biology study, supporting the hybrid origin of the polyploids in the CC population.

At the DNA-band level, there were three DNA fragments (200, 600,1900bp) of the Sox genes in RCC and 2nCC, and four (200, 500, 600, 1900bp) in 3nCC and 4nCC (Figure 6). The 500bp band found in 3nCC and 4nCC could not be amplified in 2nCC and RCC. The 2nCC was the original member in the CC population. Thus, the amplified 500bp fragments exhibited the variety in DNA sequences of polyploids. We considered the variations in genome DNA of 3nCC and 4nCC were either directly inherited from other species outside of the population or produced by genetic recombination of high probability that resulted from distant crossing.

At the DNA sequence level, the grouping of mt-DNA sequences of different ploidy CC supported the view that the CC population possessed the same maternal parent in their evolution. The *Sox *genes are located at euchromosome in the nucleus, which were highly conserved in animals. However, we found many mutations in the 1900-bp fragments of Sox4 gene in different forms of CC. Besides, in the phylogenetic study, the division of the 1900-bp sequences between polyploid CC and 2nCC indicated that there was recombination during the formation of polyploid CC. This was strong evidence of the hybrid origin of 3nCC and 4nCC. In addition, considering the possible hybrid origin and the homogenous characteristics of the polyploid CC, genomic silencing was also indicated.

## Conclusions

Hybridization played an important role in the process of polyploidization, which not only brought in a heterogenous genome, but also resulted in microchromosomes, which are of great importance in biological adaptability and in maintaining the gene communion within the population, and even in the whole process of biologic evolution.

We further presumed that the polyploidy of CC originated from hybridization was probably due to human activity. CC was miniature fish, living in certain territories. Populations of CC of different localities were diversified by the isolation. The wild crucian carp in China used to be detected as all diploid form; however, the triploid forms came to public sight since 1980s. In our opinion, together with the economic development of China and the increasing human activities since 1980s, hybridization turned up among populations of wild crucian carp that used to be diversified. Analogously, individuals of high ploidy have been discovered for a long time in another triploid Ginbuna, *Carassius auratus *langsdorfii (*Cyprinidae, Pisces*) [41]. It seemed that due to the earlier economic development of Japan, the polyploidization of crucian carp was caused by hybridization and occurred earlier in Japan and later in China. Hybridization brought in polyploidization, and form polyploid individuals. Thus, this kind of polyploidization was indirectly resulted from human activities. Human activities would finally result in the change of fish ploidy and influence evolution process.

Consequently, this is the first case about discovery of tetraploid CC together with diploid and triploid forms in a natural population in China. The series of breeding tests and molecular and cellular analyses of the population provided valuable information. It was rather extraordinary discovery that 3nCC was bisexual fertile. Substantial evidences pointed to the hybrid origin of the polyploid CC. In a word, the natural CC population we obtained in this study possesses great significance for the study of polyploidization and the evolution of vertebrates.

## Methods

### Source of samples

During 2005 to 2009, the sampled CC samples were captured with townet from Xiangjiang River, Zijiang River, Yuanjiang River, Lishui River and Dongting Lake of the Dongting water system, Hunan province. The ploidy of each sample was confirmed by detecting DNA content of blood corpuscle. The different ploidy forms were separately bred in the Engineering Research Center of Polyploid Fish Breeding and Reproduction of State Education Ministry located in Hunan Normal University. Other fish samples, including red crucian carp (RCC), common carp and blunt snout bream were collected from the Engineering Research Center of Polyploid Fish Breeding and Reproduction of State Education Ministry in Hunan Normal University.

### Measurement of DNA content

To detect the ploidy of fish samples, a flow cytometer (Partec GmbH) was employed for DNA content measurement. Red blood cells were collected from the caudal vein into syringes containing approximately 200-400 units of sodium heparin. The blood samples (5-10 μl of each fish) were treated with DAPI DNA staining solution for 10-15 min, and then filtered. The DNA content of diploid red crucian carp was used as a control. White spermatic fluid samples were stripped out from mature males of 3nCC, and then treated and tested as above. The measurement of DNA content made it possible to test the ploidy in a fast and accurate way with the least harm to the samples.

### Observation of the chromosomes of CC kidney cells

To determine the ploidy, five individuals of each ploidy form that were previously determined by DNA content were randomly sampled. Further chromosome counts were done on kidney tissue of samples. After cultured for 1-3 days at the water temperature of 22°C, the samples were injected with Phytohemagglutinin (PHA) two times at a dose of 10 μg/g body weight. The interval time of injection was 12-24 h. 2-6 hours prior to dissecting, each sample was injected with colchicine at a dose of 2-4 μg/g body weight. The kidney tissue was ground in 0.8% NaCl, followed by hypotonic treatment with 0.075 M KCl at 37°C for 40-60 min and then fixed in 3:1 methanol-acetic acid with three times. Two to three drops of cell soliquoid were dropped onto cold, wet slides and dried over a flame, then stained for 1 h in Giemsa. Chromosome metaphases were observed and photographed with Pixera Pro 600ES (US). For each group of different ploidy, 200 good-quality metaphase spreads were analyzed.

### Measurement of morphological traits

To detect the appearance diversity in the population, 15 individuals of each ploidy form that were previously determined by DNA content were randomly sampled for morphologically examining. The countable traits were examined including the number of dorsal fins, lateral scales, and upper and lower lateral scales. The measurable traits included the average values of the whole length (WL), the body length (BL) and width (BW), the head length (HL) and width (HW), and the tail length (TL) and width (TW). Some measurable data were changed into ratios.

### Observation of the ovarian microstructure

Five female individuals of each ploidy form that were previously determined by DNA content were randomly sampled. The ovaries of different ploidy CC were fixed in Bouin's solution for preparation of tissue sections. Paraffin-embedded sections were cut and stained with hematoxylin and eosin. Ovarian structure was observed with a Pixera Pro 600Es microscope.

### Observation of the testis ultra-structure using TEM

Five male individuals of 2nCC and 3nCC that were previously determined by DNA content were randomly sampled. The testis of mature male individuals of 2nCC and 3nCC were fixed in 3% glutaraldehyde solution, transferred into osmic acid solution after being washed by phosphate buffer, and finally dehydrated by an acetone gradient and embedded in Epon812. Ultrathin sections were cut and stained with uranyl acetate and lead citrate. A Hitachi-600 electron microscope was used to observe and photograph the ultra-structure of the samples.

### Observation of the sperm ultra-structure using SEM

Five male individuals of 2nCC and 3nCC that were previously determined by DNA content were randomly sampled. Samples of white spermatic fluid were stripped from 2nCC and 3nCC during the reproductive season. The samples were fixed in 3% glutaraldehyde solution for 4-10 h, and dropped onto slides after being washed in phosphate buffer three times. The samples were then transferred into 1% osmic acid solution for 1 h, dehydrated by an acetone gradient after being washed with phosphate buffer. Following this, they were immersed in tertiary butyl alcohol for 2 h, desiccated and gilded by iron sputtering. An X-650 (HITA) SEM electron microscope was employed to observe the ultrastructure of the samples. The spermatozoa head volume was calculated by (4/3) πR^2^, where R is the radius of the sperm head.

### Breeding tests

The eggs produced by 2nCC, 3nCC and 4nCC were fertilized with different species of distant relationships, including intraspecific crossing, intergeneric crossing (with *Cyprinus carpio*), inter-subfamily crossing (with *Megalobrama amblycephala*), and crossing with UV-irradiated spermatozoa of blunt snout bream *(Megalobrama amblycephala)*. The milt of male blunt snout bream were stripped, diluted with Hank's solution (1:4), and then poured into cold culture dishes to allow it to form a thin layer (0.1-0.2 mm thick). The culture dishes were put on ice and exposed to two UV lamps of 15W at a distance of 10 cm. The irradiation lasted for 22-31 min with a continuous shaking. The irradiation was monitored by the vitality of spermatozoa under a microscope. After UV irradiation, spermatozoa were kept in glass tubes and stored at 4°C.

The progenies of each crossing were bred separately, and then their morphological traits and gonadal structure were observed. The ploidy of every progeny was detected by DNA content. Chromosome counts on kidney tissues were done on five progenies of each form of different ploidy which were randomly sampled from each crossing.

### DNA Markers derived from the HMG-box of Sox genes

Degenerate primers [42] were used to amplify whole genome DNA:

P (+): 5'-TGAAGCGACCCATGAA(C/T) G-3';

P (-): 5'-AGGTCG (A/G) TACTT (A/G) TA (A/G) T -3'.

The polymerase chain reaction (PCR) amplifications were carried out in 25 μl final volume containing 80 ng DNA, 200 μM of each dNTP, 0.25 μM of each primer and 1.25 unit of *Taq*-polymerase. Cycle parameter was as follows: 94°C for 5 min; 35 cycles at 94°C for 30 s, 50°C for 45 s, 72°C for 80 s; and a final extension of 10 min at 72°C. The GeneAmp^® ^PCR System 2700 thermal cycler was employed. Amplification products were separated by electrophoresis through 1.0% agarose gel. The selected specific 1900bp bands were excised and purified from the gel using a DNA Gel Extraction Kit (Sangon), and ligated into the pMD18-T vector (TakaRa). Positive clones were identified by blue/white colony screening and PCR amplification, and then sequenced on an automated DNA sequencer (ABI PRISM 3730). For each group, over five individuals were sequenced. The nucleotide sequence alignment is executed of the Sox gene by CLUSTALW2 (http://www.ebi.ac.uk/Tools/clustalw2/).

### Sequence analysis of the complete mitochondrial DNA

PCR primers (Table 8) were designed based on the complete mitochondrial DNA (mtDNA) sequences of *Cyprinidae *fishes retrieved from GenBank. PCR amplification was carried out in 50 μL reaction mixture containing 10-30 ng DNA, 5 μL 10 × *Pfu *Buffer, 1.5-2.0 mM MgCl_2_,0.4 μM of each primer, 0.2 mM dNTP, and 1.2U *Pfu Taq *DNA polymerase(TaKaRa). PCR conditions were 94°C for 4 min, 30 cycles at 94°C for 45 s, 50-58°C for 60 s, 72°C for 1-2 min, and a final extension at 72°C for 10 min. PCR products were directly sequenced using the primer walking method on an ABI 377 automatic sequencer. All sequences were analyzed using Blast (http://www.ncbi.nlm.nih.gov), CLUSTALW (2.0), and MEGA 4.0 programs to determine the identity.

**Table 8:** Primer combinations for amplifying the complete mitochondrial sequences

Primers	Nucleotide sequence (5'→3')	Primers	Nucleotide sequence (5'→3')
cytb-F1	AATGACTTGAAGAACCACCGT	cytb-F2-2	TTCTTTCCACCCATACTTT
cytb-R1	CTCCGATCTTCGGATTACAAGAC	cytb-R2-2	AGGAACCAGATGCCAGTA
CR-F	ACCCCTGGCTCCCAAAGC	12SrRNA-F	ACAAAGCATAGCACTGAAGATG
CR-R	ATCTTAGCATCTTCAGTG	12SrRNA-R	TTTGCATGGATGTCTTCTCG
12SrRNA-R-R1	GAAACAGTGCTTGAAGGAGG	16SrRNA-F2	CGCCTGTTTACCAAAAACATCG
16SrRNA-F2-R1	AGCCCTCGTTTAGCCATT	16SrRNA-R2	CCGGTCTGAACTCAGATCA
ND1-F	TCGACGAGGGGGTTTACGAC	ND2-2F	TCCTGGTGCTTCCTTTAC
ND1-R	AGTAGTTCCTAGTCCTAGGC	ND2-2R	AGCGGTTCCTACTATTCC
ND2-2R-F	GGCCTCGATCCTACAAAC	COI-F2	TCCTCCTTCTCCTATCACT
COI-F2-F	CGTCCATTCCGACAGTAA	COI-R2	CTGGGACTGCGTCTATTT
COII-F	AATTGCTCTACCATCCCT	ATPase-F	AAAGCGTTGGCCTTTTAAGC
COII-R	GCTCATTTATGTCCTCCT	CR-F-F	AAGGAGGACCCAAGAACG
CoNd3-2F	CTAAGCCTATACCTACAAGAA	ND4-2F	TTTCTTTACGCTTCTTCC
CoNd3-2R	AACATAAGAGTGCGGAGA	ND4-2R	GCAAATTGACCCTGTTAT
ND56-2F-2	GCTACACTTATCCCAACC	ND56-2R-2-F	TGCCCTCTATGTAACCTGAT
ND56-2R-2	TAACCCGATGTCTCCTAC	ND56-4F-F	TTCCTCGTGAGATGTCGT
ND56-4F	CTAGCCAACATGACGAGC		
ND56-4R	TGAGAATGCGGTTGAAAT		

### Phylogenetic analysis

The published sequences of complete mtDNA were obtained from the NCBI GenBank. The accession numbers were: *Megalobrama amblycephala *(blunt snout bream, [GenBank: EU434747.1]), *Cyprinus carpio *(common carp, [GenBank:AP009047.1]), *Danio rerio *(zebrafish, [GenBank:NC_002333.2]), *Carassius cuvieri *(Japanese crucian carp, [GenBank: AB045144.1]), *Carassius carassius red var. *(red crucian carp, [GenBank: AY714387]). The published 1900-bp Sox fragment of red crucian carp (RCC, 1958bp) was also employed with the accession no. [GenBank: EF219275]).

The phylogenetic tree was constructed on the complete sequences of mtDNA using neighbor-joining (NJ) program of MEGA (4.0) software package based on the Kimura 2-parameters model. The statistical reliability was tested using bootstrap support (BS). The BS values of nodes of the subtree were obtained after 1000 replicates.

## List of abbreviations

2nCC: diploid crucian carp; 3nCC: triploid crucian carp; 4nCC: tetraploid crucian carp; CC: crucian carp; RCC: red crucian carp; 4nAT: tetraploid hybrids of red crucian carp (♀) × common carp (♂); 3nRB: triploid hybrids of red crucian carp (♀) × blunt snout bream (♂); 4nRB: tetraploid hybrids of red crucian carp (♀) × blunt snout bream (♂)

## Authors' contributions

JX and TZ designed of the study took charge of sample sourcing, morphological or some cytological experiments and wrote the final drafts of the paper. YC, MT, YL, CZ and LC contributed to the molecular experiments and results analysis. RZ was very conscientious in DNA content measurement. CY provided much kind help in sequences analysis of mitochondrial DNA. YZ and JY were in charge of fish breeding and crossing. Professor SL and YL provided academic advising of this study. All authors read and approved the final manuscript.

## Supplementary Material

Additional file 1 **The weight and measurable traits of 2nCC**. Click here for file

Additional file 2 **The countable traits of 2nCC**. Click here for file

Additional file 3 **The weight and measurable traits of 3nCC**. Click here for file

Additional file 4 **The countable traits of 3nCC**. Click here for file

Additional file 5 **The weight and measurable traits of 4nCC**. Click here for file

Additional file 6 **The countable traits of 4nCC**. Click here for file

Additional file 7 **The fertilization rate and hatching rate of different crossing**. Click here for file

Additional file 8 **Sequence homology analysis**. The sequences of 1900-bp fragments obtained in 2nCC, 3nCC and 4nCC were compared. Click here for file
