# Supplementary material for: Coexistence of diploid, triploid and tetraploid crucian carp (Carassius auratus) in natural waters
Source: BMC Genet. 2011 Jan 29;12:20. doi: 10.1186/1471-2156-12-20 (PMC3040159; doi:10.1186/1471-2156-12-20)
Supplement: Additional file 1 — The weight and measurable traits of 2nCC. [file 1471-2156-12-20-S1.DOC]

Table 1 The weight and measurable traits of 2nCC

| No. | Weight(g) | Whole length (cm) | Body length (cm) | Body width (cm) | Head length (cm) | Head width (cm) | Tail length (cm) | Tail width (cm) |
| --- | --- | --- | --- | --- | --- | --- | --- | --- |
| 1 | 18 | 10 | 8.1 | 2.8 | 2.1 | 2.0 | 1.1 | 1.2 |
| 2 | 22 | 10.1 | 7.9 | 3.1 | 2.2 | 2.0 | 1.3 | 1.6 |
| 3 | 10 | 8.8 | 7.6 | 2.7 | 2.0 | 1.8 | 1.0 | 1.2 |
| 4 | 9 | 8.0 | 6.3 | 2.6 | 1.8 | 2.0 | 1.0 | 1.1 |
| 5 | 14 | 9.8 | 7.5 | 2.6 | 2.2 | 2.1 | 1.4 | 1.3 |
| 6 | 13 | 9.4 | 7.1 | 3.0 | 2.0 | 1.8 | 1.2 | 1.2 |
| 7 | 6 | 7.3 | 6.3 | 2.2 | 1.5 | 1.4 | 0.9 | 1.0 |
| 8 | 7 | 8.2 | 6.6 | 2.6 | 1.6 | 2.0 | 1.1 | 1.3 |
| 9 | 25 | 11.0 | 8.9 | 3.5 | 2.1 | 2.0 | 1.3 | 1.6 |
| 10 | 9 | 8.5 | 6.7 | 2.5 | 2.0 | 1.7 | 1.3 | 1.5 |
| 11 | 15 | 10.1 | 8.0 | 2.8 | 2.1 | 1.7 | 1.5 | 1.4 |
| 12 | 9 | 8.2 | 6.5 | 2.5 | 1.7 | 1.9 | 1.0 | 1.2 |
| 13 | 7 | 8.0 | 6.7 | 2.2 | 1.8 | 1.5 | 0.9 | 1.2 |
| 14 | 12 | 9.5 | 7.9 | 2.8 | 2.1 | 2.0 | 1.5 | 1.5 |
| 15 | 17 | 10.3 | 8.3 | 2.9 | 2.3 | 2 | 1.9 | 1.5 |
