# Supplementary material for: Coexistence of diploid, triploid and tetraploid crucian carp (Carassius auratus) in natural waters
Source: BMC Genet. 2011 Jan 29;12:20. doi: 10.1186/1471-2156-12-20 (PMC3040159; doi:10.1186/1471-2156-12-20)
Supplement: Additional file 2 — The countable traits of 2nCC. [file 1471-2156-12-20-S2.DOC]

Table 2 The countable traits of 2nCC

| No. | No. of lateral scales | No. of upper lateral scales | No. of lower lateral scales | No. of dorsal fins |
| --- | --- | --- | --- | --- |
| 1 | 29 | 6 | 6 | Ⅲ+17 |
| 2 | 29 | 6 | 6 | Ⅲ+18 |
| 3 | 28 | 6 | 6 | Ⅲ+18 |
| 4 | 28 | 6 | 6 | Ⅲ+16 |
| 5 | 27 | 6 | 6 | Ⅲ+17 |
| 6 | 28 | 6 | 6 | Ⅲ+17 |
| 7 | 28 | 6 | 6 | Ⅲ+18 |
| 8 | 28 | 6 | 6 | Ⅲ+16 |
| 9 | 30 | 7 | 6 | Ⅲ+17 |
| 10 | 28 | 6 | 6 | Ⅲ+16 |
| 11 | 28 | 5 | 6 | Ⅲ+17 |
| 12 | 29 | 5 | 6 | Ⅲ+17 |
| 13 | 28 | 6 | 6 | Ⅲ+17 |
| 14 | 29 | 6 | 6 | Ⅲ+17 |
| 15 | 29 | 6 | 6 | Ⅲ+16 |
