# Supplementary material for: Coexistence of diploid, triploid and tetraploid crucian carp (Carassius auratus) in natural waters
Source: BMC Genet. 2011 Jan 29;12:20. doi: 10.1186/1471-2156-12-20 (PMC3040159; doi:10.1186/1471-2156-12-20)
Supplement: Additional file 7 — The fertilization rate and hatching rate of different crossing. [file 1471-2156-12-20-S7.DOC]

Table 7 The fertilization rate and hatching rate of different crossing

| Crossing | Total no. of eggs | No. survival to the blastula stage | No. of fry | Fertilization rate | Hatching rate |
| --- | --- | --- | --- | --- | --- |
| 2nCC（♀）×2nCC（♂） | 945 | 794 | 672 | 84% | 71% |
| 2nCC（♀）×3nCC（♂） | 1433 | 674 |  | 47% |  |
| 2nCC（♀）×common carp（♂） | 845 | 660 | 556 | 78% | 66% |
| 2nCC（♀）×blunt snout bream（♂） | 702 | 323 | 119 | 46 | 17 |
| 2nCC（♀）×UV-irradiated blunt snout bream（♂） | 1086 | 381 |  | 35% |  |
| 3nCC（♀）×2nCC（♂） | 2464 | 2146 | 1897 | 87% | 77% |
| 3nCC（♀）×3nCC（♂） | 1724 | 1428 | 1197 | 83% | 69% |
| 3nCC（♀）×common carp（♂） | 1330 | 1208 | 1145 | 91% | 86% |
| 3nCC（♀）×blunt snout bream（♂） | 913 | 823 | 594 | 90% | 65% |
| 3nCC（♀）×UV-irradiated blunt snout bream（♂） | 1108 | 872 | 751 | 79% | 68% |
| 4nCC（♀）×2nCC（♂） | 734 | 527 | 470 | 72 | 64 |
| 4nCC（♀）×3nCC（♂） | 941 | 721 | 630 | 77 | 67 |
| 4nCC（♀）×common carp（♂） | 670 | 540 | 477 | 81 | 71 |
| 4nCC（♀）×blunt snout bream（♂） | 785 | 622 | 547 | 79 | 70 |
| 4nCC（♀）×UV-irradiated blunt snout bream（♂） | 577 | 406 | 364 | 70 | 63 |
