# Supplementary material for: Coexistence of diploid, triploid and tetraploid crucian carp (Carassius auratus) in natural waters
Source: BMC Genet. 2011 Jan 29;12:20. doi: 10.1186/1471-2156-12-20 (PMC3040159; doi:10.1186/1471-2156-12-20)
Supplement: Additional file 8 — Sequence homology analysis. The sequences of 1900-bp fragments obtained in 2nCC, 3nCC and 4nCC were compared. [file 1471-2156-12-20-S8.DOC]

Table 8 Sequence homology analysis. The sequences of 1900-bp fragments obtained in 2nCC, 3nCC and 4nCC were compared

CLUSTAL 2.0.11 multiple sequence alignment

2nCC TGAAGCGACCCATGAACGCAAACCGCAATGACGCTTTCAGTCGCATTATTTCGCTGATGT

4nCC TGAAGCGACCCATGAACGCAAACCGCAATGACGCTTTCAGTCGCATTATTTCGCTGATGG

RCC TGAAGCGACCCATGAACGCAAACCGCAATGACGCTTTCAGTCGCATTATTTCGCTGATGT

3nCC TGAAGCGACCCATGAACGCAAACCGCAATGTCGCTTTCAGTCGCATTATTTCGCTGATGG

****************************** ****************************

2nCC ACAGTCAAGTGAGGAGCGTGACATTATTCCAAAGATCTCACGCTTGAGCTCATGTTTTGG

4nCC ACAGTCAAGTGAGGAGCGTGACATTATTCCAAAGAGCTCACGCTTGAGCTCATGTTTTGG

RCC ACAGTCAAGTGAGGAGCGTGACATTATTCCAAAGATCTCACGCTTGAGCTCATGTTTTGG

3nCC ACAGTCAAGTGAGGAGCGTGACATTATTCCAAAGATCTCACGCTTGAGCTCATGTTTTGG

*********************************** ************************

2nCC GTAAACAATAACAAGGATGTAAGCAAACCGTTGAGAAAGATCTGTCGGCTGGTCGTTATG

4nCC GTAAACAATAACAAGGATGTAAGCAAACCGTTGAGAAAGATCTGTCGGTTGGTCGTTATG

RCC GTAAACAATAACAAGGATGTAAGCAAACCGTTGAGAAAGATCTGTCGGTTGGTCGTTATG

3nCC GTAAACAATAACAAGGATGTAAGCAAACCGTTGAGAAAGATCTGTCGGTTGGTCGTTGTG

************************************************ ******** **

2nCC TCATTCTTAAACAAATATAGATTCTGATGCATTAACAGCAGGCGAGCTGCGCTTGGCTGC

4nCC TCATTCTTAAACAAATATAGATTCTGATGCATTAACAGCAGGCGAGCTGCGCTTGGCTGC

RCC TCATTCTTAAACAAATATAGATTCTGATGCATTAACAGCAGGCGAGCTGCGCTTGGCTGC

3nCC TCATTCTTAAACAAATATAGATTCTGATGCATTAACAGAAGGCGAGCTGCGCTTGGCTGC

************************************** *********************

2nCC ACTCTGCCATCTAGCGTCACTGCCGTCCAGTGTGTCCTCGGCGGGTGTGGACCAGGGGCA

4nCC ACTCTGCCATCTAGCGTCACTGCCGTCCAGTGTGTCCTCGGCGGGTGTGGACCAGGGGCA

RCC ACTCTGCCATCTAGCGTCACTGCCGTCCAGTGTGTCCTCGGCGGGTGTGGACCAGGGGCA

3nCC ACTCTGCCATCTAGCGTCACTGCCGTCCAGTGTGTCCTCGGCGGGTGTGGACCAGGGGCA

************************************************************

2nCC GGGCTCTGTTCGGGTCCCGGTCAAAACATGCACCGGTGTCTATGAAGCACTGGCGCGCGG

4nCC GGGCTCTGTTCGGGTCCCGGTCAAAACATGCACCGGTGTCTATGAAGCACTGGCGCGCGG

RCC GGGCTCTGTTCGGGTCCCGGTCAAAACATGCACCGGTGTCTATGAAGCACTGGCGCGCGG

3nCC GGGCTCTGTTCGGGTCCCGGTCAAAACATGCACCGGTGTCTATGAAGCACTGGCGCGCGG

************************************************************

2nCC GCAGCAGGGGTAAGACGTGACGTACGACACAAACTTGTTCCAGCGCCTTCAGCGCCTTAA

4nCC GCAGCAGGGGTAAGAGGTGACGTACGACACAAACTTGTTCCAGCGCCTTCAGCGCCTTAA

RCC GCAGCAGGGGTAAGACGTGACGTACGACACAAACTTGTTCCAGCGCCTTCAGCGCCTTAA

3nCC GCAGCAGGGGTAAGAGGTGACGTACGACACAAACTTGTTCCAGCGCCTTCAGCGCCTTAA

*************** ********************************************

2nCC AGATCTCTCAGAATCTGGAGAGCGCTGCTAGCCTCGAACTCTGCTG------CGTCTTCT

4nCC AGATCTCTCAGAATCTGGAGAGCGCTGCTAGCCTCGAACTCTGCTGCTGTTGCGTCTTCT

RCC AGATCTCTCAGAATCTGGAGAGCGCTGCTAGCCTCGAACTCTGCTACTGTTGCGTCTTCT

3nCC AGATCTCTCAGAA-------------------CTCGAACTCTGCTGCTGTTGCGTCTTCG

************* ************* *******

2nCC GATTGGGGCGTGCGCTCTGTGACGAAGGACCGCCCAGCTGCTCAGAGGCCCCGCCCTCTC

4nCC GATTGGGGCGTGCGCTCTGTGACGAAGGACCGCCCAGCTGCTCAGAGGCCCCGCCCTCTC

RCC GATTGGGGCGTGCGCTCTGTGACGAAGGACCGCCCAGCTGCTCAGAGGCCCCGCCCTCTC

3nCC GTTTGGGGCGTGCGCTCTGTGACGAAGGACCGCCCAGCTGCTCAGAGGCCCCGCCCTCTC

* **********************************************************

2nCC CCGAACCAGCCAAAACAACGCGCATAGCCTGAACTCACTCACTCACACTGAGCCTCGATC

4nCC CCGAACCAGCCAAAACAACGCGCATAGCCTGAACTCACTCACTCACACTGAGCCTCGATC

RCC CCGAACCAGCCAAAACAACGCGCATAGCCTGAACTCACTCACTCACACTGAGCCTCGATC

3nCC CCGAACCAGCCAAAACAACGCGCATAGCCTGAACTCACTCACTCACACTGAGCCTCGATC

************************************************************

2nCC CTGCCACTGCACACAAAAACAGCCCTGAGCACGAGACATGAGCGAGAGCTTGATGGCAGA

4nCC CTGCCACTGTTCACCGACACAGCCCTGAGCACGAGACATGAGCGAGAGCTTGATGGCAGA

RCC CTGCCACAGTTCACCGACACAGCCCTGAGCACGAGACATGAGCGAGAGCTTGATGGCAGA

3nCC CTGCCACTGTTCACCGACACAGCCCTGAGCACGAGACATGAGCGAGAGCTTGATGGCAGA

******* * *** * ******************************************

2nCC TATTGATTAATATAGCCTACACAGAAAGTCCATTTAAAGCTTTCCATTATTCATGGGCAG

4nCC TATTGATTAATATAGCCTACACAGAAAGTCCATTTAAAGCTTTCCATTATTCATGGGCAG

RCC TATTGATTAATATAGCCTACACAGAAAGTCCATTTAAAGCTTTCCATTATTCATGGGCAG

3nCC TATTGATTAATATAGCCTACACAGAAAGTCCATTTAAAGCTTTCCATTATTCATGGGCAG

************************************************************

2nCC GATTCTTTCATTCATTTTGATGTATTGGCTATTTCATGTTGTTTTGTTTCTTTACATCTA

4nCC GATTCTTTCATTCATATTGATGTATTGGCTATTTCATGTTGTTTTGTTTCTTTGCATCTA

RCC GATT----CATTCATTTTGATGTATTGGCTATTTCATGTTGTTTTGTTTCTTTACATCTA

3nCC GATT----CTTTCATTTTGATGTATTGGCTATTTCATGTTGTTTTGTTTCTTTGCATCTA

**** * ***** ************************************* ******

2nCC TAAGTGCATGTCAGAATGCAGTCTGTTATGAACATATCCAGATTTGTTTTCTAACTCAAA

4nCC TAAGTGCATGTCAGAATGCAGTCTGTTATGGACATATCCAGATTTGTTTTCTAACTCAAA

RCC TAAGTGCATGTCAGAATGCAGTCTGTTATGAACATATCCAGATTTGTTTTCGAACTCAAA

3nCC TAAGTGCATGTCAGAATGCAGTCTGTTATGAACATATCCAGATTTGTTTTCTAACTCAAA

****************************** ******************** ********

2nCC AGCAGAGTGTGTTTTT-ATTTCTAAGGATTAAAAGATCACGCCTCCCTTATCGACATGCA

4nCC AGCAGAGTGTGTTTTT-ATTTCTAGGGATTAAAAGATCACGCCTCCGTTATCGACATGCA

RCC AGCAGAGTGTGTTTTT-ATTTCTAAGGATTAAAAGATCACGCCTCCCTTATCGACATGCA

3nCC AGCAGAGTGTGTTTTTTATTTCTAAGGATTAAAAGATCACGCCTCCCTTATCGACATGCA

**************** ******* ********************* *************

2nCC AAAATAAAATAAAAATACACATCAGCGTGTTTCATTAAAACTAAGTTTGTCGTGTACACT

4nCC AAAATAAAATAAAAATACACATCAGCGTGTTTCATTAAAACTAAGTTTGTCGTGTACACT

RCC AAAATAAAATAAAAATACACATCAGCGTGTTTCATTAAAACTAAGTTTGTCGTGTACACT

3nCC AAAATAAAATAAAAATACACATCAGCGAGTTTCATTAAAACTAAGTTTGTCGTGTACACT

*************************** ********************************

2nCC GGAATGAGGTCAGACCCAAAGCTCCTCATTGCACGCGCTGATGATTATTAGATTCCTTTT

4nCC GGAATGAGGTCAGGCCCAAAGCTCCTCATTGCACGCGCTGATGATTATTAGATTCCTTTT

RCC GGAATGAGGTCAGACCCAAAGCTCCTCATTGCACGCGCTGATGATTATTAGATTCCTTTT

3nCC GGAATGAGGTCAGACCCAAAGCTCCTCATTGCACGCGCTGATGATTATTAGATTCCTTTT

************* **********************************************

2nCC TGCGCCAATGAAAAGAGAGTGGGCGTGTTTTTGGCTCGGGGAAGGGTAGAGACAGCAGCT

4nCC TGCGCCAATGAAAAGAGAGTGGGCGTGTTTTTGGCTCGGGGAAGGGTAGAGACAGCAGCT

RCC TGCGCCAATGAAAAGAGAGTGGGCGTGTTTTTGGCTCGGGGAAGGGTAGAGACAGCAGCT

3nCC TGCGCCAATGAAAAGAGAGTGGGCGTGTTTTTGGCTCGGGGAAGGGTAGAGACAGCAGCT

************************************************************

2nCC CATTGGCCGGCCGCCGTCGCTATTATACCATTGGAAACGGATAAAACTGTATGAAGCAGA

4nCC CATTGGCCGGCCGCCGTCGCTATTATACCATTGGAAACGGATAAAACTGCATGAAGCAGA

RCC CATTGGCCGGCCGCCGTCGCTATTATACCATTGGAAACGGATAAAACTGCATGAAGCAGA

3nCC CATTGGCCGGCCGCCGTCGCTATTATACCATTGGAAACGGATAAAGCTGCATGAAGCAGA

********************************************* *** **********

2nCC ATAGAAGTCTGTTGAAACTTACAGACTGTTTTCAAGAGACGAACACTTGTGCTAGCATCA

4nCC ATAGAAGTCTGTTGAAACTTACAGACTGTTTTCAAGAGACGAACACTTGTGCTAGCAACA

RCC ATAGAAGTCTGTTGAAACTTACAGACTGTTTTCAAGAGACGAACACTTGTGCTAGCATCA

3nCC ATAGAAGTCTGTTGAAACTTACAGACTGTTTTCAAGAGACGAACACTTGTGCTAGCATCA

********************************************************* **

2nCC CATCCCGGTTTTCCCGCATGGTTTGAACTGGGAAGGATATGCAGAAGGAGTCAGATATCT

4nCC CATCCCGGTTTTCACGCATGGTTTGAACGGGGAAGGATATGCAGAAGGAGTCAGATATCT

RCC CATCCCGGTTTTCCCGCATGGTTTGAACGGGGAAGGTTATGCAGAAGGAGTCAGATATCT

3nCC CATCCCGGTTTTCCCGCATGGTTTGAACTGGGAAGGATATGCAGAAGGAGTCAGATATCT

************* ************** ******* ***********************

2nCC TCCGAAACTCGAACAGAATACACACTAACACTGAGCCCTCGCACCACTGCGCTCATATAC

4nCC TCCGAAACTCGAACAGAATACACACTAACACTGAGCCCTCGCACCACTGCGCTCATATAC

RCC TCCGAAACTCGAACAGAATACACACTAACACTGAGCCCTCGCACCACTGCGCTCATATAC

3nCC TCCGAAACTCGAACAGAATACACACTAACACTGAGCCCTCGCACCACTGCGCTCATATAC

************************************************************

2nCC AAACTTTCAACTGCTCTTCGACTGTTACAGCATTATTACTGGATGCACGGACACTGTGGA

4nCC AAACTTTCAACTGCTCTTCGACTGTTACAGCATTATTACTGGATGCACGGACACTGTGGA

RCC AAACTTTCAACTGCTCTTCGACTGTTACAGCATTATTACTGGATGCACGGACACTGTGGA

3nCC AAACTTTCAACTGCTCTTCGACTGTTACAGTATTATTACTGGATGCACGGACACTGTGGA

****************************** *****************************

2nCC CATCGGACACTGTATCAGCTCCGTGAACGGTGTGATGAAGCCACGCCACGGCACTAAGCA

4nCC CATCGGACACTGTATCAGCTCCGTGAACGGTGTGATGAAGCCACGCCACGGCACTAAGCA

RCC CATCGGACACTGTATCCGCTCCGTGAACGGTGTGATGAAGCCACGCCACGGCACTAAGCA

3nCC CATCGGACACTGTATCCGCTCCGTGAACGGTGTGATGAAGCCCCGCCACGGCACTAAGCA

**************** ************************* *****************

2nCC CGACTGAAGCGTGTGGTGCACCAGCTCTCCTCAGGACTACTATCAGACTGAGTTTTCTTT

4nCC CGACTGAAGCGTGTGGTGCACCAGCCCTCCTCAGGATTACTATCAGACTGAGTTTTCTTT

RCC CGACTGAAGCGTGTGGTGCACCAGCTCTCCTCAGGACTACTATCAGACTGAGTTTTCTTT

3nCC CGACTGAAGCGTGTGGTGCACCAGCTCTCCTCAGGACTACTATCAGACTGAGTTTTCTTT

************************* ********** ***********************

2nCC AGCGACACGGTGGACTTGAGAGCGCACAGAGGCTCTCTC----TC----------ACACA

4nCC AGCGACACGGTGGACTTGAGAGCGCACAGAGGCTCTCTCACTCTC----------ACACA

RCC AGCGACACGGTGGACTTGAGAGCGCACAGAGGCTCTCTCTCTCTCTC---TACACACACA

3nCC AGCGACACGGTGGACTTGAGAGCGCACGGAGGCTCTCTCTCTCTCTCACACACACACACA

*************************** *********** ** *****

2nCC CACACAGACACACACACAC----AGACTGGTGTAGCAATGGTCCAGAAAACAAGCAGCAC

4nCC CACACACACACACACACACTCACAGACTGGTGTAGCAATGGTCCAGAAAACAAGCAGCAC

RCC CACACACACACACACACACTCACAGACTGGTGTAGCAATGGTCCAGAAAACAAGCAGCAC

3nCC CACACACACACACACACACACACAGACTGGTGTAGCAATGGTCCAGAAAACAAGCAGCAC

****** ************ *************************************

2nCC GGTGTTTGCTGGGGACTCGTCCGATTCCGGCGCGCTGGATCTGGACATGGCTTCTTCCCC

4nCC GGTGTTTGCTGGGGACTCGTCCGATTCCGGCGCGCTGGATCTGGACATGGCTTCTTCCCC

RCC GGTGTTAGCTGGGGACTCGTCCGATTCCGGCGCGCTGGATCTGGACATGGCTTCTTCCCC

3nCC GGTGTTTGCTGGGGACTCGTCCGATTCCGGCGCGCTGGATCTGGACATGGCTTCTTCCCC

****** *****************************************************

2nCC GACCCCCGGCTCCACGGCATCCGGGGGTGACAAGCTTGACCCCGGCTGGTGCAAAACCCC

4nCC GACCCCCGGCTCCACGGCATCCGGGGGTGACAAGCTTGACCCCGGCTGGTGCAAAACCCC

RCC GACCCCCGGCTCCACGGCATCCGGGGGTGACAAGCTTGACCCCGGCTGGTGCAAAACCCC

3nCC GACCCCCGGCTCCACGGCATCCGGCGCGGACAAGCTTGACCCCGGCTGGTGCAAAACCCC

************************ * ********************************

2nCC CAGCGGTCACATCAAGAGACCGATGAACGCGTTCATGGTGTGGTCGCAAATCGAGAGGCG

4nCC CAGCGGTCACATCAAGAGACCGATGAACGCGTTCATGGTGTGGTCACAAATCGAGAGGCG

RCC CAGCGGTCACATCAAGAGACCGATGAACGCGTTCATGGTGTGGTCGCAAATCGAGAGGCG

3nCC CAGCGGTCACATCAAGAGACCGATGAACGCGTTCATGGTGTGGTCTCAAATCGAGAGGCG

********************************************* **************

2nCC CAAGATCATGGAGCAGTCACCGGACATGCACAACGCGGAGATCTCCAAGAGACTCGGCAA

4nCC CAAGATCATGGAGCAGTCACCGGACATGCACAACGCGGAGATCTCCAAGAGACTCGGCAA

RCC CAAGATCATGGAGCAGTCACCGGACATGCACAACGCGGAGATCTCCAAGAGACTCGGCAA

3nCC CAAGATCATGGAGCAGTCACCGGACATGCACAACGCGGAGATCTCCAAGAGACTCGGCAA

************************************************************

2nCC GCGCTGGAAGCTCCTCAAAGACAGCGACAAGATCCCGTTCATCCGAGAGGCGGAGCGCCT

4nCC GCGCTGGAAGCTCCTCAAAGACAGCGACAAGATCCCGTTCATCCGAGAGGCGGAGCGCCT

RCC GCGCTGGAAGCTCCTCAAAGACAGCGACAAGATCCCGTTCATCCGAGAGGCGGAGCGCCT

3nCC GCGCTGGAAGCTCCTCAAAGACAGCGACAAGATCCCGTTCATCCGAGAGGCGGAGCGCCT

************************************************************

2nCC GCGGCTCAAGCACATGGCAGACTACCCCGACTACAAGTACCGACCT

4nCC GCGGCTCAAGCACATGGCAGACTACCCCGATTACAAGTACCGACCT

RCC GCGGCTCAAGCACATGGCAGACTACCCCGACTACAAGTACCGACCT

3nCC GCGGCTCAAGCACATGGCAGACTACCCCGACTACAAGTATCGACCT

****************************** ******** ******
